# Halo Nevi Are Not Trivial: About 2 Young Patients of Regressed Primary Melanoma That Simulates Halo Nevi

**DOI:** 10.1155/2021/6672528

**Published:** 2021-03-22

**Authors:** S. De Schrijver, I. Theate, O. Vanhooteghem

**Affiliations:** ^1^Dermatology Department, CHU UCL Namur, Site Sainte Elisabeth, Place Louise Godin 15, 5000 Namur, Belgium; ^2^Institute of Pathology and Genetics (IPG), Avenue George-Lemaître 25, 6041 Gosselies, Belgium

## Abstract

**Background:**

Halo nevi are often considered benign, and the possibility of malignancy is not always clear to practitioners. We present two case reports suggesting that a halo nevus appearance can be seen in melanoma, even in young adults. A literature search for halo nevi revealing melanoma shows that this is a very rare condition. *Case presentation*. This report of two young patients indicates the importance of obtaining a detailed history to detect warning signs such as itching, pain, spontaneous bleeding, and previous alterations according to the patient, including a previously totally black colour in an already fully regressed melanoma.

**Conclusions:**

The risk of a halo nevus being malignant is higher if there is only one unique halo nevus and no personal or familial history of vitiligo. We postulate that a regressing atypical nevus or a regressing melanoma may be induced by an immunologic reaction as halo nevus type of clinical picture.

## 1. Introduction

Halo nevus, also called Sutton's nevus, is a melanocytic nevus surrounded by a halo of depigmentation, which is usually symmetrically round or oval. Halo nevi affect up to 5% of Caucasian children six to fifteen years old [1, 2] in an equal sex distribution and are more common in patients with an increased number of nevi and/or a personal or familial history of vitiligo. Halo nevus is seen in common acquired nevi, though it may be seen in a congenital nevus, blue nevi, Spitz nevi, and even, but rarely, in melanoma. The most common location is the back, and multiple lesions are present in approximately half of all cases [2–4]. This regression of pigmentation is believed to present a T-cell mediated immune response to nevus antigens [3]. There are four clinical stages in the life of a halo nevus, with the duration of the process ranging from months to decades [4, 5]. Starting as a pigmented nevus surrounded by a halo of depigmentation (stage 1), a pink nevus surrounded by a halo of depigmentation then develops (stage 2), followed by a circular area of depigmentation with total or partial disappearance of the nevus (stage 3) and finally normal-appearing skin after repigmentation of the halo (stage 4). It is important to assess the clinical features of the halo nevus [6]. A biopsy is not indicated if the pigmented part is regular in appearance; however, biopsy might be justified in the presence of atypical or worrisome features, as a halo appearance can be seen in dysplasia or, rarely, in melanoma.

## 2. Case Presentation 1

**Figure 1:**
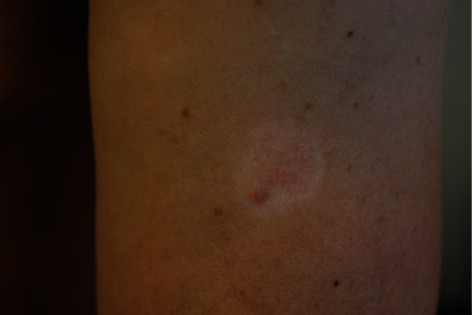
Halo nevus on the left upper arm. The central part shows only a light shadow while the rest of the lesion is depigmented.

**Figure 2:**
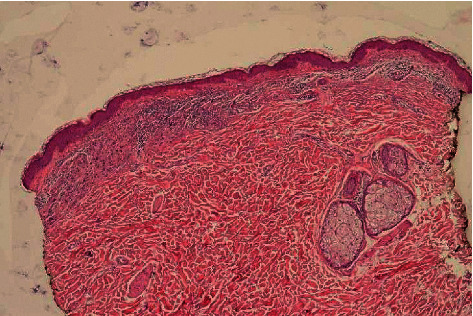
Intradermal atypical nevus cells in the left part of the biopsy, contrasting with regression in the right part (HE 5x).

**Figure 3:**
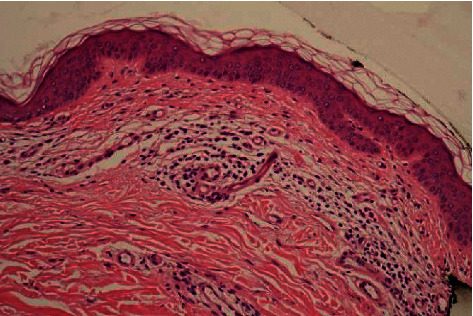
Signs of extensive regression in the superficial dermis, lacking atypical melanocytes (HE 20x).

A 43-year-old man without medical history presented with an existing irregular halo and depigmented macule on the left upper arm for two and a half years that had changed according to the patient (Figure 1). He mentioned that the lesion was first a dark black colour and then, all of a sudden, started to disappear, with currently only a light shadow remaining centrally around a further fully depigmented lesion. Clinical assessment did not reveal other halo nevi or clinical signs of vitiligo but revealed numerous subcutaneous nodular lesions disseminated over the body, particularly on the back, suggesting the possibility of metastatic disease. Histology of the halo nevus showed intradermal atypical nevus cells but also extensive regression in the superficial dermis (Figures 2 and 3). Histology of the nodular lesion on the back confirmed skin metastases of a melanoma. The suspicion of a regressing melanoma with diffuse metastases (stage IV) with a V600E mutation in the BRAF gene was confirmed. PET-CT demonstrated diffuse metastases near the lungs, adrenal glands, ganglions, peritoneum, retroperitoneum, pericardium, and brain. The patient was referred to oncology for further assessment and therapy but die quickly.

## 3. Case Presentation 2

**Figure 4:**
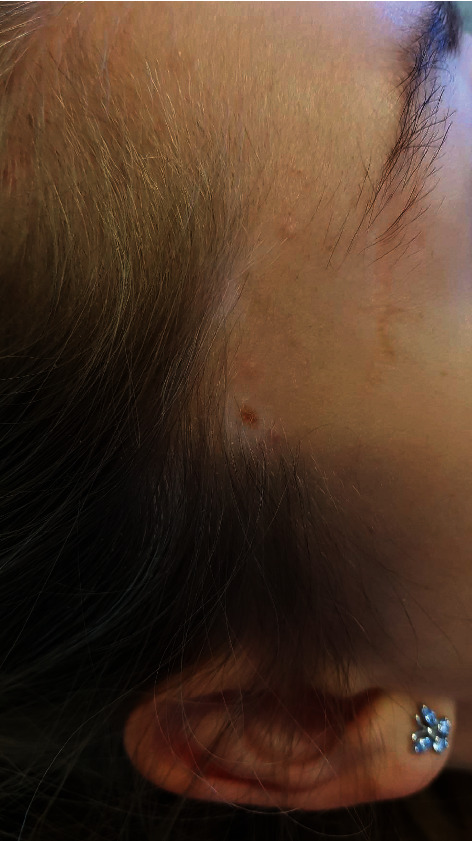
Nevus on the right temporal area surrounded by a symmetrical rim of depigmentation.

**Figure 5:**
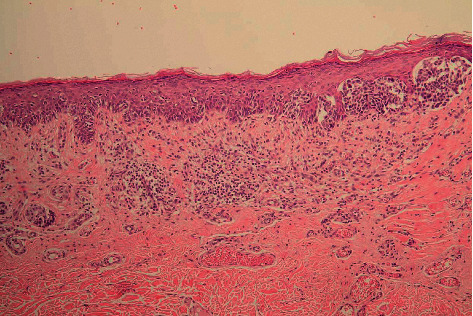
The lesion is characterized by atypical junctional activity and signs of extensive regression in the superficial dermis (HE × 10).

**Figure 6:**
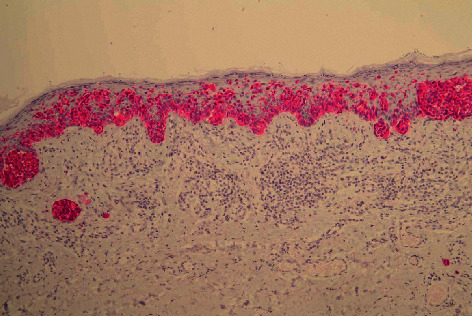
Immunohistochemistry shows bridging and pagetoid spread of atypical melanocytes (Melan A red × 10).

A 21-year-old girl without medical history mentioned having a congenital nevus on the right temple that had started to itch some weeks ago. Clinical evaluation revealed a nevus with a halo of depigmentation measuring six by eight millimetres, with a central zone of homogenic pigmentation (Figure 4). Clinical assessment did not reveal other halo nevi or other diseases, such as vitiligo. Dermatoscopy showed some telangiectasias surrounded by a symmetrical rim of depigmentation. Although there was no clear suspicion of malignancy, excision was performed because the patient was anxious. Histology showed a melanocytic lesion with extensive regression at the margins of the lesion with only a few atypical cells in the dermis (Figure 5). In contrast, the centre of the lesion showed dense melanocytic proliferation with severe atypical features in the dermoepidermal junction as well as in the dermis (Figures 6). This picture confirmed the diagnosis of a superficial spreading melanoma, Breslow 0.70 mm, Clark level three, but no mitoses, ulcerations or invasion into the lymphatics, vasculature, or nerves (pT1a) were observed. Re-excision with large margins was carried out. Follow-up showed no recurrence.

## 4. Discussion

There is still much discussion about the origin of halo nevi and whether they should be regarded as benign or as a possible reaction to an atypical nevus or melanoma. Weyant et al. sought to determine the frequency of dysplastic nevi and other histologic types of nevi in a series of halo nevi that were diagnosed clinically [7]. They found that out of the 124 halo nevi that were reviewed, 48 (38.7%) were dysplastic nevi, and only 3 (2.4%) were melanomas. Mooney et al. reviewed 142 halo nevi [8]. For 66 cases, the diagnosis of halo nevus was made both clinically and pathologically, and for 76 cases, the diagnosis was based on histological results alone. Among those with a clinicopathological diagnosis of halo nevus, 11% exhibited moderate atypia, 16% exhibited minimal atypia to only focally moderate atypia, 24% exhibited minimal atypia, and 49% exhibited no significant atypia. Among patients diagnosed only by pathology, there was also a broad spectrum of atypia, with 8% exhibiting focally severe or severe atypia. This study supports the concept that the halo nevus should not be regarded as a single clinicopathological entity but rather a wide spectrum of histological atypia. For these 142 cases of halo nevi, the authors could not find histology revealing a melanoma. It is important to distinguish halo nevi from regressing melanoma. Therefore, investigators are looking for differences in histopathology between halo nevi seen in benign lesions versus those seen in regressing melanoma. The main differences between the histopathological appearance of a halo nevus and melanoma can be summarized as follows: in the former, nevi cells are arranged in nests, while in melanoma, there are isolated atypical melanocytes in the epidermis and aggregates in the dermis. There is also a well-known difference between the symmetric appearance of nevi and the asymmetric appearance of melanoma. Mature cells with rare or a lack of mitosis are characteristic of a nevus when compared with the numerous mitotic immature cells in melanoma. Even so, in halo nevi, there is a diffuse lymphocytic infiltration throughout the entire lesion, in contrast to melanoma, where the inflammatory infiltrate is concentrated in the periphery. Nevi cell destruction, characteristic of the biological evolution of the halo nevi, has not resulted in the development of fibrosis [9]. On the other hand, the destruction of tumour cells during the regression of melanoma results in papillary dermal fibrosis during the final stage of resorption. Considering all the above, we can state that a depigmented halo must be regarded as a phenomenon that may be associated with different types of melanocytic tumours and with a broad spectrum of histopathological atypia degree. Therefore, investigators should also look for specific characteristics both clinically and dermatoscopically to predict whether the halo nevus is benign or malignant. Blessing et al. considered that halos associated with melanoma have a more asymmetric shape than those associated with melanocytic nevi [9]. Kolm et al. studied the dermatoscopic characteristics of halo nevi and found that the characteristic dermatoscopic features of benign melanocytic nevi, represented by globular and/or homogeneous patterns, are typically observed in children and young adults [10]. However, halo nevi revealed considerable changes in area over time during digital dermoscopic follow-up, albeit their structural patterns remained unchanged. For this reason and because melanoma with halo-like depigmentation, although rare, additionally exhibits melanoma-specific dermoscopic criteria, the authors concluded that the role of digital dermoscopic follow-up in the diagnosis of halo nevi is nonsignificant. Regarding patient age, Rubegni et al. established that patients who have a halo associated with melanoma are older than patients who have a halo nevus associated with benign melanocytic nevi [11]. As our patients are only 21 and 43 years old, we cannot confirm this point of view, and we stress that young age cannot definitively classify the halo nevus as benign. Further studies are expected to add valuable information regarding the features of tumours with depigmented halos. Our opinion, however, based on our cases, is that obtaining a detailed history to look for signs of malignancy is at least as important as clinical and dermatoscopic examinations and therefore cannot be ignored. Halo nevi can also be observed in combination with vitiligo, although the question of whether halo nevi should be considered as a sign of vitiligo or as a risk factor for developing vitiligo is still under debate [12]. Reported incidence of halo nevi in patients with vitiligo ranges between 1% and 48% [4, 13, 14]. Van Geel et al. observed the presence of halo nevi in 31.1% of all patients with vitiligo [12]. They also found that patients with halo nevi only were less likely to experience the Koebner phenomenon than patients with vitiligo only or patients with both halo nevi and vitiligo. Zhou et al. confirmed this conclusion and statistically showed that the presence of the Koebner phenomenon in halo nevi patients is highly suggestive of an increased risk of developing vitiligo. The Koebner phenomenon is often considered a sign of more active or extensive conditions [15]. Therefore, the authors recommend that genetically susceptible individuals avoid any physical or mechanical disturbances, such as scratching or friction on melanocytic nevi, which might lead to the risk of the Koebner phenomenon and subsequent specific immune targeting of melanocytes. Considering all of the above, the concept of a halo around a nevus can be thought of a “pseudo-Koebner” phenomenon. In conclusion, halo nevi are common, especially in children and young adults. Halo nevi develop from a benign acquired melanocytic nevus but can also be seen in dysplastic nevi and even, rarely, in melanoma, which should be emphasized to more to clinicians. Young age does not automatically mean that the halo nevus is of benign origin. Further investigations are necessary to detect clearer-cut clinical and dermatoscopic features to distinguish between benign and malignant origins in halo nevi. However, in our experience, a detailed clinical history and clinical examination (e.g., itchiness and previous dark black pigmentation) are important and can already provide diagnostic clues, especially when there is only one clinical halo nevus. Biopsy is necessary in these cases to avoid missing melanoma, but confocal microscopy can help clinicians to make the decision between excision–-biopsy or follow-up [16].

## Data Availability

The data used to support the findings of this study are available from the corresponding author upon request.
